# Effects of Ionizing Radiation and Long-Term Storage on Hydrated vs. Dried Cell Samples of Extremophilic Microorganisms

**DOI:** 10.3390/microorganisms10010190

**Published:** 2022-01-16

**Authors:** Ida Romano, Carlo Camerlingo, Lisa Vaccari, Giovanni Birarda, Annarita Poli, Akira Fujimori, Maria Lepore, Ralf Moeller, Paola Di Donato

**Affiliations:** 1 Institute of Biomolecular Chemistry, National Research Council of Italy, Via Campi Flegrei, 34, 80078 Pozzuoli, Italy; ida.romano@icb.cnr.it (I.R.); annarita.poli@icb.cnr.it (A.P.); 2 SuPerconducting and Other INnovative Materials and Devices Institute, National Research Council of Italy, Via Campi Flegrei, 34, 80078 Pozzuoli, Italy; carlo.camerlingo@spin.cnr.it; 3 Elettra—Sincrotrone Trieste S.C.p.A. S.S., 14 km 163,5 in Area Science Park, Basovizza, 34149 Trieste, Italy; lisa.vaccari@elettra.eu (L.V.); giovanni.birarda@elettra.eu (G.B.); 4 Molecular and Cellular Radiation Biology Group, Department of Charged Particle Therapy Research, Institute for Quantum Medical Science, Chiba 263-8555, Japan; fujimori.akira@qst.go.jp; 5 Dipartimento di Medicina Sperimentale, Università della Campania “L. Vanvitelli”, Via S. Maria di Costantinopoli 16, 80138 Napoli, Italy; maria.lepore@unicampania.it; 6 German Aerospace Center (DLR e.V.), Institute of Aerospace Medicine, Radiation Biology Department, Aerospace Microbiology, DLR, Linder Höhe, D-51147 Köln, Germany; ralf.moeller@dlr.de or; 7 Natural Sciences Department, University of Applied Sciences Bonn-Rhein-Sieg (BRSU), von-Liebig-Straße 20, D-53359 Rheinbach, Germany; 8 Department of Science and Technology, Parthenope University of Naples, Centro Direzionale—Isola C4, 80143 Napoli, Italy

**Keywords:** extremophiles, space radiation environment, biochemical fingerprinting, STARLIFE project, SERS, IR microspectroscopy

## Abstract

A main factor hampering life in space is represented by high atomic number nuclei and energy (HZE) ions that constitute about 1% of the galactic cosmic rays. In the frame of the “STARLIFE” project, we accessed the Heavy Ion Medical Accelerator (HIMAC) facility of the National Institute of Radiological Sciences (NIRS) in Chiba, Japan. By means of this facility, the extremophilic species *Haloterrigena hispanica* and *Parageobacillus thermantarcticus* were irradiated with high LET ions (i.e., Fe, Ar, and He ions) at doses corresponding to long permanence in the space environment. The survivability of HZE-treated cells depended upon either the storage time and the hydration state during irradiation; indeed, dry samples were shown to be more resistant than hydrated ones. With particular regard to spores of the species *P. thermantarcticus*, they were the most resistant to irradiation in a water medium: an analysis of the changes in their biochemical fingerprinting during irradiation showed that, below the survivability threshold, the spores undergo to a germination-like process, while for higher doses, inactivation takes place as a consequence of the concomitant release of the core’s content and a loss of integrity of the main cellular components. Overall, the results reported here suggest that the selected extremophilic microorganisms could serve as biological model for space simulation and/or real space condition exposure, since they showed good resistance to ionizing radiation exposure and were able to resume cellular growth after long-term storage.

## 1. Introduction

An investigation of the limits of life is a key issue in astrobiology, either for the search of life on other planets or for the identification of terrestrial organisms that potentially could adapt to living beyond Earth. The environmental conditions of space and exoplanets constitute a big challenge for life as we know it, since they are considered “extreme” on both chemical and physical points of view. The space radiation is a main environmental factor that strongly limits the possibility of life outside of Earth due to its lethal effects: living organisms potentially travelling through space or living on the surface of planets lacking the protection that terrestrial atmosphere and magnetic field give, are exposed to the harmful effects of the radiation field. The space environment of our solar system is indeed characterized by a complex radiation field that includes the solar components (i.e., photons and protons with low-medium energy) and the Galactic Cosmic Rays (GCRs) originating from astrophysical events taking place outside the solar system but generally inside our galaxy [1,2]. The GCRs are composed of high-energy protons, α-particles, and high atomic number nuclei and energy (HZE) ions that in turn represent about 1% of GCRs and are the major cause of the lethal effects of this radiation. The biological effects produced by an HZE have been extensively studied in a variety of space experiments or on ground simulation facilities using the biological dosimeter spores of *Bacillus subtilis* species [2,3,4,5]. These studies showed that the ionizing radiation effects depend upon both the delivered dose and the type of radiation; indeed, the LET value directly correlates with the number of changed cell structures and molecules that are passed through the particle’s path [2,3,4,5]. Fe, Ar, and He ions are high-LET particles (>2 keV/mm), and they can be harmful by means of two different processes, i.e., “direct hits’’ and ‘‘indirect hits’’: in the first case, the cell is in the track core of the HZE particle, and it is passed via the particle; in the second case, the cell is hit only by high-energy secondary electrons, i.e., δ-rays, which are produced by interaction of the HZE with the biological target. Cells undergoing direct hits will likely be killed, while cells hit by the so-called δ-rays will survive most probably by producing mutants [2]. Moreover, when radiation passes through a biological target, two types of interaction can take place: a direct radiation effect, i.e., direct energy absorption by DNA, proteins, and other cellular components; an indirect radiation effect, i.e., interaction of cellular targets with radicals generated by radiolysis of water, i.e., the so-called reactive oxygen species (ROS) [2,4], or other chemical species [2]. In January 2018, we had the opportunity to participate in the “STARLIFE” project [4], an international intercomparison radiation campaign that studied the effect of ionizing radiations on different biological model systems relevant to astrobiology [3,4]. To identify new microbial species that potentially could resist space radiation and to test microorganisms that could be used for real space exposure, we proposed using two extremophilic microorganisms belonging to both the Archaea and Bacteria domains that, in our previous studies [6,7,8], showed promising resistance to multiple extreme parameters mimicking the space environment as biological samples. The selected species were the archaeon *Haloterrigena hispanica*-type strain FP1T [9], an extremely halophilic species with optimal growth at 50 °C, and the thermophilic species *Parageobacillus thermantarcticus* [8], a bacterium isolated from an active volcano in Antarctica, which already showed good resistance to ionizing radiations [8].

The effects of the irradiation process have been studied by analyzing the survival probability and the cells’ response at a molecular level. Survival assays were carried out soon after the irradiation while the changes occurring in the molecular composition of the spores and the environmental fluid have been investigated by Surface-Enhanced Raman Spectroscopy (SERS) and Infra-Red (IR) spectroscopy.

The radiation doses used for this study allowed for simulating long-lasting exposure to space radiation; therefore, to assess if cells could resume their ability to restart growth long after HZE treatment, the survival assay coupled with the analysis of molecular modifications were repeated one year after the irradiation campaign. Finally, the effect of water, the absence of which is a main factor characterizing space and exoplanets environments, was taken also in account: indeed, as previously reported for the radioresistant species *Deinococcus radiodurans* [10,11], the hydration state can strongly affect the ionizing radiations’ effects. Therefore, HZE exposure was carried out for all microorganisms on either hydrated or desiccated cells and spores.

## 2. Materials and Methods

### 2.1. Production and Purification of Spores of Parageobacillus Thermantarcticus

Spores of *Parageobacillus thermantarcticus* strain M1 were produced as previously described [8]. In brief, sporulation was induced by inoculating the strain in 100 mL (1:100 *v*/*v*) of the sporulating medium YNM (0.6% yeast extract (Oxoid, Themofisher, Waltham, MA USA 02451), 0.3% NaCl (Merck KGaA, Darmstadt, Germany), and 0.001% MnSO_4_ (Merck KGaA, Darmstadt, Germany); pH 5.8 in tap water). The strain was incubated at 60 °C in static conditions and the sporulation process was verified by means of microscope analysis. After 48 h of incubation, vegetative cells and spores were harvested by centrifugation at 10,000 rpm for 15 min. The resulting pellet was washed with sterile distilled water, and spores were recovered by centrifugation at 4000 rpm for 15 min. The washing treatment was repeated until the spore suspension contained 99% phase-bright spores, as confirmed by phase-contrast microscopy analysis. One milliliter of this spores’ suspensions, with a final concentration of 10^8^ CFU/mL (Colony Forming Unit), was placed in a Corning tube and stored at 4 °C until the irradiation experiments. Survivors after irradiation were determined by measuring the CFU/mL values. A same aliquot of spores was air dried and stored at room temperature for 5 years: survivors of this sample were determined by measuring the optical density (O.D.) at 600 nm of cells grown in the standard conditions, and the inoculum size was 6 mg cells/25 mL of the YN standard medium. All the experiments were run in triplicate.

### 2.2. Vegetative Cell Culture Conditions

*Haloterrigena hispanica*-type strain FP1^T^ [9] was grown in its standard conditions as previously described. For irradiation in isotonic solution (IS, composition: sodium citrate 3g/L; MgSO_4_·7H_2_O 20 g/L; NaCl 200 g/L; FeCl_2_·4H_2_O 36 mg/L; and MnCl_2_· 4H_2_O 0.36 mg/L (Merck KGaA, Darmstadt, Germany) in deionized water), 1.5 mL of each cell culture with an optical density (O.D.) value of 0.700 at 660 nm was collected and centrifuged at 10,000 rpm for 10 min. The resulting cell pellets were washed with IS (1.5 mL × 2), re-suspended in a total volume of 1 mL of IS, and stored at 4 °C for irradiation experiments. For survival assays, the inoculum size was 1:25 *v*/*v*. For air-dried cells, 25 mg of cell pellet obtained after washing with IS and lyophilization were collected and stored at room temperature before irradiation experiments. Aliquots of 5 mg were used for irradiation and then were used as an inoculum in 2 mL of medium for the survival assay. All the experiments were run in triplicate.

### 2.3. Radiation Exposure Conditions

HZE exposure experiments were carried out at the Heavy Ion Medical Accelerator (HIMAC) facility of the National Institute for Radiological Sciences (NIRS) in Chiba, Japan. The ionizing radiations were generated using accelerated ions of helium, argon, and iron at different doses and linear energy transfers (LETs). The radiation dose in the SI was measured in Grays, with 1 Gy representing the net absorption of 1 J in 1 kg of water. The LET represents the energy that a high-energy-charged (HZE) particle transfers to a biological sample when it travels through it. The irradiation geometry of the HIMAC and dose calculations have been previously described [12]. The radiation energies and LET are presented in Table 1.

### 2.4. HZE Cross Section Calculations

To compare the radiation effects due to different kinds of ions, an equivalent radiation dose can be defined by taking into account the σ cross section of the single direct elastic collision of the ion with the target atoms of irradiated molecules. Borrowing a model widely used in solid-state physics, this σ−parameter estimates the effect of a single collision event, and it can be evaluated by using Rutherford’s relation [13]:

(1) $$\sigma =\pi \frac{M_{1}{\left[Z_{1}Z_{2}e^{2}\right]}^{2}}{M_{2}E}\frac{dT}{T^{2}}$$

where T is the transferred energy from an ion of energy E, and M_i_ and Z_i_ are the mass and the atomic number of the incident ions (i = 1) and of the target (i = 2), respectively. The cross section allows for estimating the relevance of damages produced by the radiation, which are strongly dependent on the M_1_ mass and atomic number Z_1_ of the ions (see Equation (1)). For a fixed target atoms of mass M_2_ and atomic number Z_2_, the calculated relative cross-sectional magnitudes for He, Ar, and Fe ions are reported in Table 2, with values normalized to the Fe ion cross section.

### 2.5. Surface-Enhanced Raman Spectroscopy (SERS) Measurements

Raman spectroscopy is based on an inelastic scattering process of the light in which the scattered photon is shifted in frequency from the incident photon as it either loses energy to or gains energy from a particular vibrational mode of the molecule. This technique allows for estimating the fundamental vibrations of functional groups that can be employed to determine the chemical structure and dynamics of molecules of interest [14]. Based on Raman spectroscopy, Surface-Enhanced Raman Spectroscopy (SERS) represents an evolution of the conventional method that, using the surface plasmon properties of metallic nanoparticles, allows for a large enhancement of the sensitivity. SERS was implemented by gold nanoparticles (GNPs) with 26 nm diameters that allows for an optimal SERS response for the He–Ne laser excitation source used. GNPs were obtained by a chemical process based on a citrate reduction of HAuCl_4_ [15]. Close to the GNP metallic surface, an enhancement of the Raman signal is expected from the substance to probe. A Raman signal enhancement of the order of 10^3^ was obtained for Rhodamine 6G/water solutions using a concentration as low as 5 μM [16]. For the present series of measurements, a small amount of the colloid containing the GNPs was added to a 1 μL drop of the spore suspension, and slightly reshuffled by using the pipette tips. The drop was left to dry on a microscope cover glass for almost half an hour into a laminar flow cabinet before the SERS acquisition. A micro-Raman system Jobin-Yvon equipped with a TRIAX 180 monochromator and a liquid-nitrogen-cooled CCD detector was used. A spectral resolution of 4 cm^−1^ was obtained. The 17 mW He–Ne laser beam was focused on the sample by a 50X (n.a. 0.75) optical objective using an Olympus BX40 microscope. Times of 60 s were typically employed for SERS acquisition.

### 2.6. Synchrotron Infrared Microspectroscopy

Once received, the samples were vortexed to re-suspend the pellet possibly formed, and 2 drops of 5 μL were deposited on top of the other onto a ZnSe window, the second after the first dried. Single point measures were carried out with an IR–Vis microscope, Bruker’s Hyperion 3000 ((Bruker Corporation, 40 Manning Road, Billerica, MA, USA), equipped with Mercury Cadmium Telluride (MCT) single-point detector, setting the apertures at 30 × 30 to map few spores per time and averaging 512 scans (scanner speed 120 kHz) with a spectral resolution of 4 cm^−1^ in the 650–4000 cm^−1^ spectral range. More than one hundred spectra were collected for each treatment.

Once acquired, the data were corrected for the atmospheric contribution with OPUS 7.5 (Bruker Optics) and saved. Further analyses were performed with Quasar (quasar.codes) [17,18]; by using this software, the data were first preprocessed with baseline correction, smoothed by 15 points with a Savitzky–Golay filter, vector normalized over the whole range and cut in the range of interest 1800–950 cm^−1^, and then analyzed with a Principal Component Analysis (PCA). The results were plotted using Origin 2020 (Origin Labs). Band integrals were calculated, drawing a linear baseline between the extremes of each range of interest: 1735–1480 cm^−1^ for proteins, 1700–1600 cm^−1^ for amide I, 1480–1330 cm^−1^ for Ca^2+^-DPA, and 1180–950 cm^−1^ for carbohydrates. Ratios, averages, and standard deviations were calculated using Origin 2020 (Origin Labs) software.

## 3. Results

### 3.1. Microorganisms’ Survival to Irradiation with HZE

Irradiation of cell samples was carried out with ions at different LETs, energies, and delivered doses, as reported in Table 1 (Section 2.3).

For all of the selected species, two kinds of cell samples were irradiated: dry powdered cells/spores and cells/spores suspended in isotonic solution/water, respectively. The resistance of the selected extremophilic species was evaluated upon receiving the samples from Chiba Laboratories, i.e., 1 month after irradiation experiments; the measure was then repeated 1 year after storage at 4 °C.

**Figure 1:**
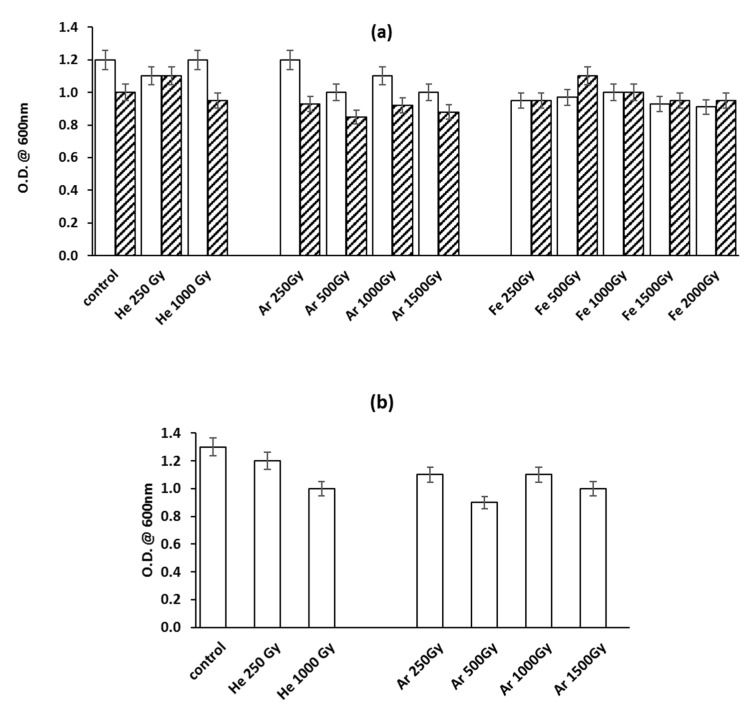
*Haloterrigena hispanica*’s vegetative cells survival after HZE irradiation: (**a**) dried cells irradiated with He, Ar, and Fe ions; (**b**): cells in isotonic solution, irradiated with He and Ar ions (legend: O.D.: optical density at 660 nm; open bars: O.D. 1 month after irradiation; dashed bars: O.D. 1 year after irradiation).

HZE irradiation of the halophilic species *Haloterrigena hispanica* was carried out on dry cells and vegetative cells in isotonic solution (Figure 1a,b, respectively); in both cases, the resistance of the halophile was assayed as the ability to restart growth when irradiated cells were used as inoculum in their optimal culture conditions.

*H. hispanica* dried cells resisted all HZE particles at all doses delivered (Figure 1a). He, Ar, and Fe ion irradiation did not significantly affect the growth ability of the vegetative cells, since either 1 month or 1 year after the irradiation, the final O.D. values were comparable with the control sample. The main differences were found in the growth’s kinetics; indeed, growth delays of one day, three days, and four days were found for cells irradiated with He, Ar, and Fe ions, respectively. Interestingly, dried cells were the only types able to resist HZE Fe particle irradiation: indeed, as shown in Figure 1a, the cell biomass productivity of Fe-irradiated samples was comparable with that of the control sample either 1 month or 1 year after the HZE bombardment experiments.

*H. hispanica* vegetative cells showed an appreciable resistance to irradiation in isotonic solution only with He and Ar ions: as shown in Figure 1b, vegetative cells were able to restart growth 1 month after the HZE irradiation even at the higher delivered doses. Indeed, cells irradiated by 1000 Gy for He and 1500 Gy for Ar reached O.D. values of 77% and 91% with respect to the control sample, respectively. On the other hand, the same irradiated samples stored for 1 year were not able to grow. In this case, a growth decay of 4 days was observed for both He- and Ar-irradiated cells. In the case of Fe ions, no cell growth could be detected either from samples investigated soon after the experiments or one year later.

Similar to *H. hispanica, P. thermantarcticus* irradiation was carried out for two kinds of samples: spores that were previously air dried and then stored at room temperature for 5 years and freshly prepared spores suspended in deionized water at a concentration of 10^8^ CFU/mL.

**Figure 2:**
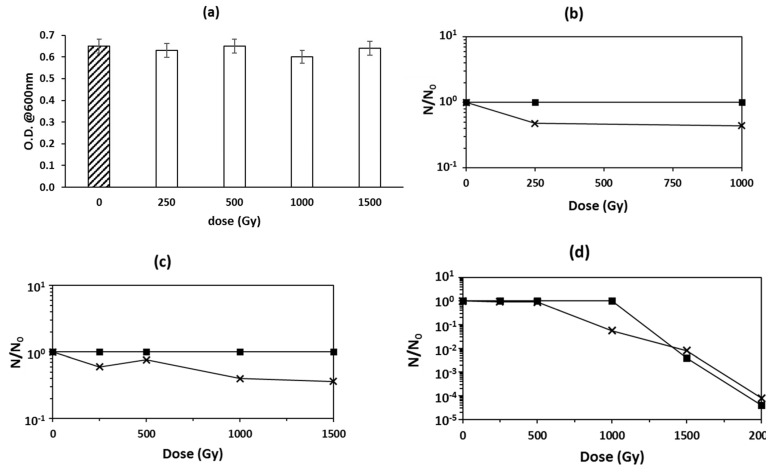
*P. thermantarcticus’* spore survival after HZE irradiation. (**a**) Dried spores irradiated with Fe ions (legend: O.D.: optical density at 660 nm); (**b**–**d**) spores irradiated in isotonic solution with He ions, Ar ions, and Fe ions, respectively. (legend: ■ spores surviving fraction measured 1 month after irradiation; × spores surviving fractions measured 1 year after irradiation).

In the case of the 5-year-old dried spores, the powdered samples were irradiated only with Fe ions at doses up to 1500 Gy and the spores’ resistance was assessed by measuring the ability of the irradiated cells to restart germination and growth, when put in the standard optimal conditions. As shown in Figure 2a, Fe ions irradiation at all doses caused no significant differences in the ability to restart growth compared with the control sample, i.e., not treated spores; on the other hand, cellular growth of irradiated cells showed a delay of one day with respect to the control sample.

In the case of freshly prepared spores, irradiation was carried out in water by means of He-, Ar-, and Fe-accelerated ions (Figure 2b–d, respectively) at doses ranging from 250 up to 2000 Gy. Spore resistance was evaluated by measuring the surviving fractions, i.e., the N/N_0_ ratio, where N was the CFU/mL of the irradiated samples while N_0_ was the value of the control samples. One month after the irradiation campaign, the CFU value of the samples treated with He and Ar ions was not affected by the HZE ions at all doses delivered (Figure 2b,c, respectively). Irradiation with Fe ions at doses up to 1000 Gy also did not affect the spore population, but a significant decrease in the surviving fraction was found at the doses of 1500 Gy and 2000 Gy, where ratios of about two and five orders of magnitude lower were observed, respectively.

The N/N_0_ measure was repeated after storage of all treated samples at 4 °C for one year in the same water medium used for HZE irradiation. As shown in Figure 2b, for the sample irradiated with He, the spore population was halved; a similar result was found also for Ar ions irradiation, which at the higher dose (1500 Gy), showed a N/N_0_ value of about 0.5. In the case of the “aged” samples treated with Fe HZE particles, the depletion of spore population was found already at 1000 Gy, where the N/N_0_ value was nearly one order of magnitude lower than the control. For higher doses, the spores’ concentration showed no significant difference with respect to that measured for the samples assayed soon after the irradiation.

### 3.2. Biological Effects of HZE on Bacterial Spores

#### 3.2.1. SERS analysis of spores of *P. thermantarcticus* after HZE ion irradiation

The effects of HZE ion irradiation on the *P. thermantarcticus* spores were also investigated at the molecular level by SERS analysis to assess changes in the biochemical fingerprinting of the bacterial spores and, eventually, released substances in the suspension medium.

**Figure 3:**
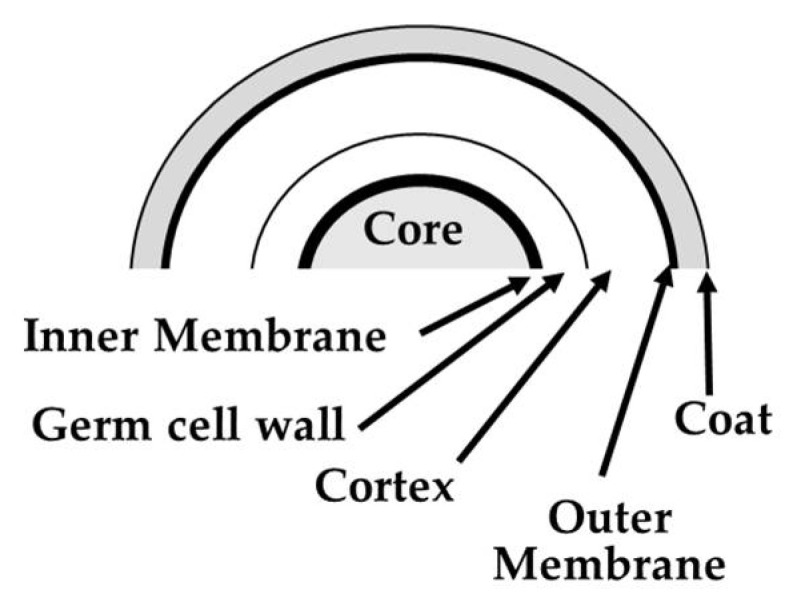
General structure of a bacterial spore.

Spores of cells belonging to the *Bacillaceae* family such as the species of *Bacillus* and related genera share a common structure that is schematically depicted in Figure 3 [16].

The inner part is represented by the core that has a low water content (25 to 50% of wet weight) and a huge amount (≈10% of total spore dry weight) of the spore-specific molecule pyridine-2,6-dicarboxylic acid (dipicolinic acid, DPA) in a 1:1 chelate with divalent cations, predominantly Ca^2+^-DPA. Spore’s DNA and proteins are suspended in this matrix of calcium dipicolinate. The core is surrounded by the inner membrane and by two peptidoglycan layers, i.e., the germ cell wall and the cortex. The latter is enclosed by an outer membrane and by the protein layer of the coat [19].

**Figure 4:**
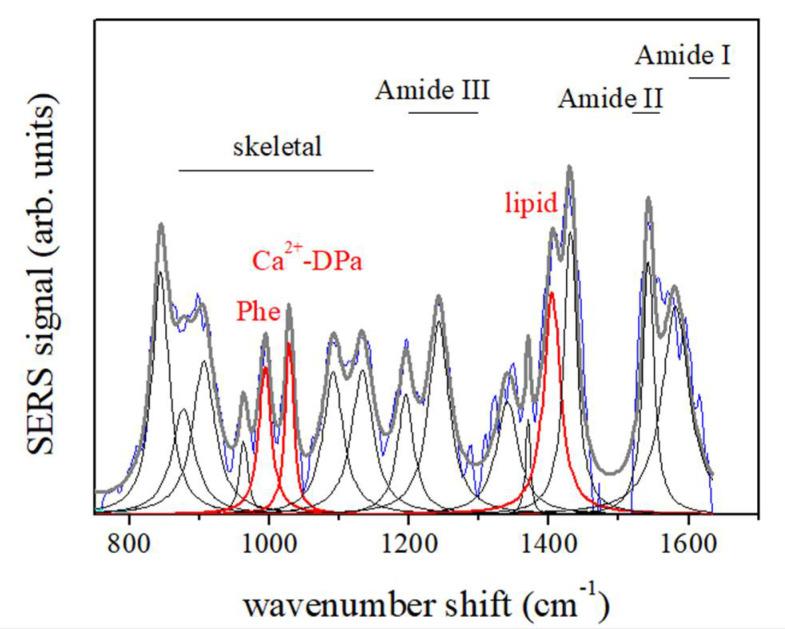
SERS response of spores *P. thermantarcticus.* This spectrum was obtained by averaging SERS spectra measured at different positions of the sample. The Lorentzian components have been determined by a fitting procedure to individuate the main modes occurring in the signal. The experimental spectrum (blue line) is compared with the fit result (grey thick line) obtained by considering the different mode components (black lines). The modes assigned to phenylalanine, Ca^2+^-DPA, and lipid contributions are reported in red.

In Figure 4, a typical SERS spectrum of not treated spores of *P. thermantarcticus* suspended in water is reported: the main signals, as previously described, have been assigned to phenylalanine at ~1000 cm^−1^, falling in the protein skeletal region ranging from 870 to 1150 cm^−1^ wave number; to lipid methylenes (CH_2_ bonds) at about 1400 cm^−1^; and to Ca^2+^-DPA mode [20] at 1024 cm^−1^ (red lines in Figure 4). The SERS signals originated from molecules that interact with the surface of metallic nanoparticles; therefore, the cell components that are on the outer shell of the spores, i.e., the coat proteins, will be responsible for the most intense signal at 1000 cm^−1^, while the resonance at 1024 cm^−1^ is attributable to the amount of released Ca^2+^-DPA. As shown by the spectrum in Figure 4, the SERS measurements performed on the control sample of spores indicated that a little Ca^2+^-DPA can be detected in the medium, also in the absence of specific triggering agents or damage processes.

The ratio between Ca^2+^-DPA and phenylalanine mode intensities (I_1025 cm_^−1^/I_1000 cm_^−1^) was then used to monitor the effects of the exposure of *P. thermantarcticus* spores to the HZE irradiation. The use of the ratio values allows for removing the artifacts due to instrumental parameters and sample variability [8,21,22,23,24]

**Figure 5:**
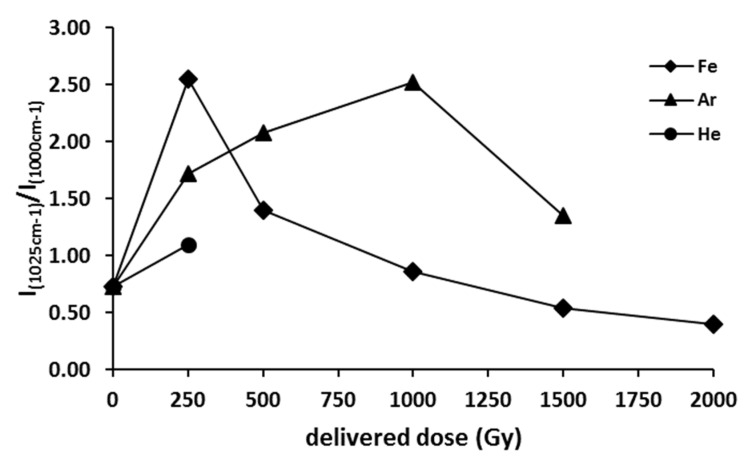
Effect of HZE irradiation on the ratio between Ca^2+^-DPA (1025 cm^−1^) and phenylalanine (1000 cm^−1^) SERS mode intensities (I_1025 cm_^−1^/I_1000 cm_^−1^) of *P. thermantarcticus*.

Irradiation with Fe HZE already at the lower dose, i.e., 250 Gy, caused an increase in the I_1025 cm_^−1^/I_1000 cm_^−1^ ratio to a maximum value (Figure 5): such a result suggested that a release of Ca^2+^-DPA from the core occurred. On the other hand, at higher doses, the ratio of Ca^2+^-DPA/phenylalanine signal intensities decreased: indeed, it reached a value comparable with the not irradiated spores at a dose of 1000 Gy, while at the highest dose (2000 Gy), it was also lower than the not irradiated sample. The trend for the I_1025 cm_^−1^/I_1000 cm_^−1^ ratio profile vs. delivered dose observed for the Ar HZE-irradiated samples (Figure 5, dashed line) was similar to that registered for the Fe experiment. Indeed, also in this case by increasing the irradiation energy, the ratio of Ca^2+^-DPA/phenylalanine signals reached a maximum value. Nevertheless, a higher radiation dose of Ar particles, in comparison with Fe HZE, was required to reach the maximum value of I_1025 cm_^−1^/I_1000 cm_^−1^.

#### 3.2.2. IR Microspectroscopy Analysis of Spores of *P. thermantarcticus* Spores after Irradiation with HZE in Solution

**Figure 6:**
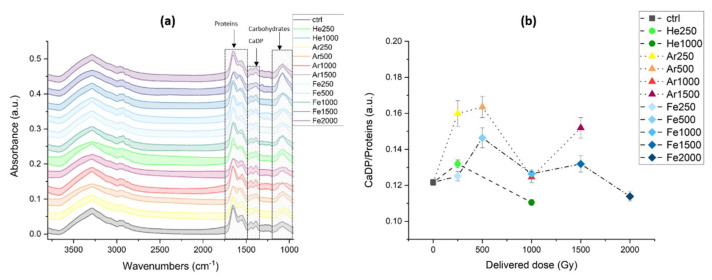
(**a**) Average spectra ± standard deviation (shaded area) of *P. thermantarcticus’* spores controls and after irradiation with different ions and energies, arrows point at the spectral ranges highlighted in light grey used to calculate the integrals shown in panel b and in Appendix A, Figure A1a,b. (**b**) scatterplot of the Ca^2+^-DPA over proteins ratio, calculated as the ratio of the area of the bands between 1480–1330 cm^−1^ over the area of the bands between 1735 and 1480 cm^−1^. For both plots, in black are the controls, in shades of green are the signals from the spores irradiated with He, in yellow-red are those irradiated with Ar, and in shades of blue are those irradiated with Fe.

**Figure A1:**
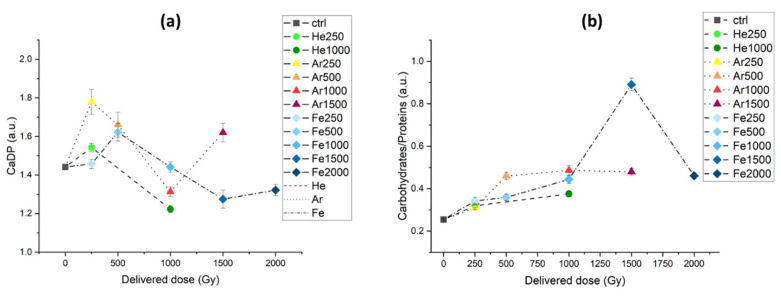
(**a**) Line plot of the Ca^2+^ -DPA signal obtained by integrating the 1480–1330 cm^−1^ spectral range. (**b**) Line plot of the ratio for carbohydrate signals, calculated as the area of the 1180–950 cm^−1^ spectral range, over the total protein signals. For all plots, different lines types and colors represent different ions, from lighter colors representing lower doses to darker colors representing higher doses**.**

The long-terms effects of HZE irradiation were also investigated by means of IR microspectroscopy, as reported in Figure 6a, where the average spectra of *P. thermantarcticus*’ spores are shown. The spectral ranges more affected by the irradiation are those in the range 1735–1480 cm^−1^, corresponding to the amide I and II protein signals, and those of the multipeak band at 1180–950 cm^−1^, conventionally assigned to phosphates (from DNA and RNA) and carbohydrates. Since the phosphates usually give rise to two main signals, one at 1230 cm^−1^ and one at 1080 cm^−1^ [25], and we see a dose-related strong increase in the broad band at 1050 cm^−1^, it is safe to assign these variations mainly to the saccharidic components of the spores. All of the ions used for the irradiation induce a strong decrease in the protein amide bands. At lower frequencies, according to the literature [26], the peaks between 1480 and 1330 cm^−1^ can be assigned to calcium dipicolinate and its several hydrated forms. Since infrared bands are quite broad and the same chemical moiety can belong to different molecules, it is not possible to be as specific at in the SERS Raman data analysis. Moreover, the intensities of the bands can be affected by the number of spores measured or their thickness; therefore, it is better to use ratios when describing the system in analysis. Thus, it is more appropriate to show and comment on the band’s intensities ratios, as shown in Figure 6b, where the ratio between the signal intensity of Ca^2+^-DPA and the proteins’ amide I + amide II has been reported as a function of increasing radiation dose. From this plot, it is possible to observe that the three ions cause slightly different modifications to the spores, which do not respond in the same way to the same dose. Exposure to helium causes a small increase in the Ca^2+^-DPA/proteins ratio at 250 Gy mainly due to the decrease in the protein band signal with respect to the Ca^2+^-DPA signal that stays constant; at 1000 Gy, the ratio decreases. The values of this ratio for argon and iron have the same trend but with different slopes and values and with the irradiation with iron ions having an additional dose at 2000 Gy, where we can notice a decrease in the ratio. Graphs similar to the one in Figure 6b are presented in Figure A1a,b in Appendix A. Figure A1a shows the variation in the Ca^2+^-DPA peaks in response to the delivered dose; it can be seen that, besides the 250 and 500 Gy irradiations, almost all signals are lower than those for the controls for He and Fe and are higher only for Ar at 1500 Gy. In Figure A1b, the ratio between the carbohydrate and the proteins is reported instead; from this plot, it is possible to observe a dose-dependent increase in these values, both due to a decrease in proteins and an increase in the polysaccharide content in the spore envelope.

**Figure 7:**
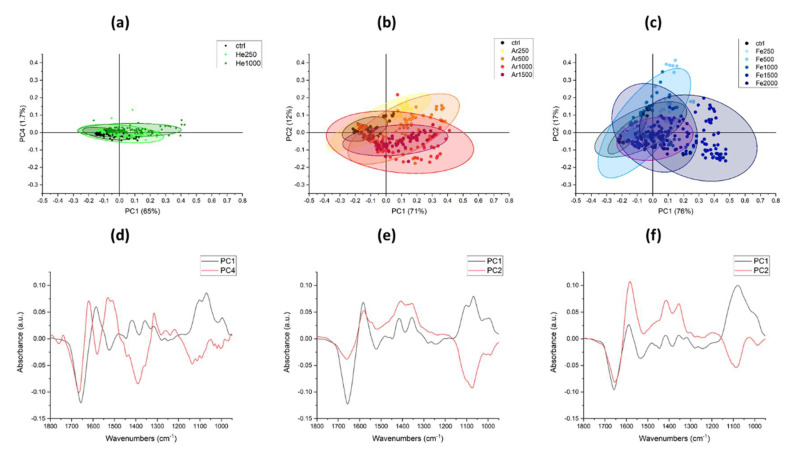
Principal components analysis (PCA) of *P. thermantarcticus’* spores’ spectra. (**a**) Scatterplot showing that, in the PC1-PC4 planes, the PCA scores of the data were acquired on the spores irradiated with He. (**b**) Scatterplot showing that, in the PC1-PC2 planes, the PCA scores of the data were acquired on the spores irradiated with Ar. (**c**) Scatterplot showing that, in the PC1-PC2 planes, the PCA scores of the data were acquired on the spores irradiated with Fe. Ellipses represent the 95% confidence value. In parenthesis, in the axis name, the percentage of variance is shown, represented by each PC (**d**–**f**), with loading vectors representing the principal components shown in panels (**a**–**c**) in the spectral range from 1800 to 950 cm^−1^.

More accurate considerations about the variations induced in the populations can be drawn by the principal component analysis outcomes, presented in Figure 7.

In panels (a) to (c) are presented the scatterplots of the scores values of the PCA in the coordinate system generated by the analytical procedure. Each dot represents one spectrum collected on a small group of spores. The plots are presented with the same scale to quickly get the grasp of the magnitude of the spectral variations induced by the treatments: for He ions, the treated spores cluster close to the controls; for Ar, the variations are bigger; and for Fe, the distribution is the broadest. The spectral features affected by the irradiation can be appreciated by looking at the loading vectors presented in Figure 7d–f. For all treatments, PC1, which is the loading that explains the most variance, is similar. Looking at the dispersion of the score spectra, their barycenter can be projected along PC1 from left to right according to the delivered dose: controls are in the negative part of the plane, and as the dose increases, the points shift towards the positive part of the plane. By assigning the peaks of PC1 to specific chemical moieties or structures, it is possible to explain the processes happening in the cells. The most intense peak is a negative signal at 1654 cm^−1^, usually assigned to the alpha conformation of proteins; hence, upon irradiation there is a decrease in these structures. The following signals of interest are the double peaks at 1445 and 1395 cm^−1^, which can be attributed to the Ca^2+^-DPA. The band at lower frequencies, 1180–950 cm^−1^, is formed by four peaks 1100, 1070, 1020, and 990 cm^−1^ and it can be assigned to the polysaccharides constituting the envelope of the spores [27,28]; it becomes more intense with an increase in the radiation dose delivered. In this spectral range, it is possible to notice the only difference between the three PC1 loadings: it is in Fe irradiation that the peaks are not as sharp as in the others, indicating a possible disorder of the saccharides’ structures.

The vertical axis of the scatterplots in Figure 7a–c represents another “direction” of variance of the systems, and the loading vectors were selected to try to obtain a separation between low doses and high doses. The loading vectors, shown in red in Figure 7d,f, vary according to the ion used to deliver the radiation. For He, PC4 was chosen, and its main signals are in the amide I range at 1660 and 1620 cm^−1^, the first negative while the latter positive, and these frequencies are usually assigned to 3_10_ alpha helices and beta sheet aggregates, respectively [29]. Regarding Ar and Fe, the PC2 loading vectors were picked and show similar spectral features, albeit of different intensity. In both, we see a negative peak in the amide I region centered at 1654 cm^−1^; then, there is the positive peaks’ doublet assigned to Ca^2+^-DPA at 1414 and 1353 cm^−1^.

## 4. Discussion

One main issue in astrobiology is the identification of terrestrial species that potentially could survive to the transfer across space or thrive on exoplanets that are characterized by extreme conditions such as a lack of protection from the ionizing radiations of GCR. In the frame of the Starlife campaign, we had the opportunity to carry out irradiation with high-LET particles (such as Fe-, Ar-, and He-accelerated ions), which were used to simulate the radiation environment experienced during space transfer or on the surface of exoplanets lacking atmosphere and magnetic fields. HZE particles can damage cells via different processes by triggering diverse cellular damages. Indeed, they can either generate ionization and excitation processes, or physicochemical processes, such as thermal spikes or shock waves. Moreover, the cellular damages can either be caused by direct absorption of energy from DNA, membranes, and protein, or by secondary events, i.e., the interaction of cellular components with reactive species such as those generated by water’s radiolysis, i.e., the so-called reactive oxygen species (ROS) [2,4]. For this study, either dried cell samples or hydrated cells (i.e., suspended in water/isotonic solutions) were used as biological models to investigate the survivability to both direct and indirect absorption of radiations. The biological samples were selected from previously studied extremophilic species, and they included either spores or viable cells.

When irradiation was carried out on dry samples, i.e., in an environment where only direct absorption of energy could take place, as previously described for other species such as *Deinococcus radiodurans* [10,11], the growth ability of either vegetative cells or spores was not completely compromised. Indeed, the cell growth for the halophilic species *H. hispanica* reached an average value of 80% with respect to the control sample (i.e., not irradiated cells) for all HZE and at all delivered doses. The survival observed 1 month after the irradiation was comparable with that measured after 1 year at 4 °C, thus suggesting that no long lasting effect of HZE irradiation was found. Similarly, dry spores of the thermophilic species *P. thermantarcticus* did not lose their ability to germinate, giving rise to cell production. This behavior is in qualitative agreement with the results reported in Ref. [3], where the influence of HZE ion irradiation on the germination process was investigated in poly-extremophilic bacilli spores. Irradiation in a water solution, for which the generation of ROS could also take place, had more significant effects on the growth ability of either cells or spores samples. In the case of vegetative cells of the halophilic species *H. hispanica*, survival was observed only 1 month after the irradiation with lower LET ions, i.e., He and Ar; indeed, Fe caused the inability to restart growth at all doses delivered. One year later, the samples that had survived He and Ar exposure completely lost their ability to restart growth. As expected, the spores of *P. thermantarcticus* showed to be more resistant than viable cells, since bacterial spores are well known to possess a structural organization that allows them to survive also in unfavorable conditions. Irradiation with higher energy doses resulted in more remarkable effects; such a phenomenon was more evident when the long-terms effects were evaluated, i.e., survivability and biochemical changes one year after the irradiation. Therefore, attention was paid to the spore samples 1 year after HZE ion irradiation in a water solution, for which SERS spectroscopy and IR microspectroscopy analyses were carried out.

**Figure 8:**
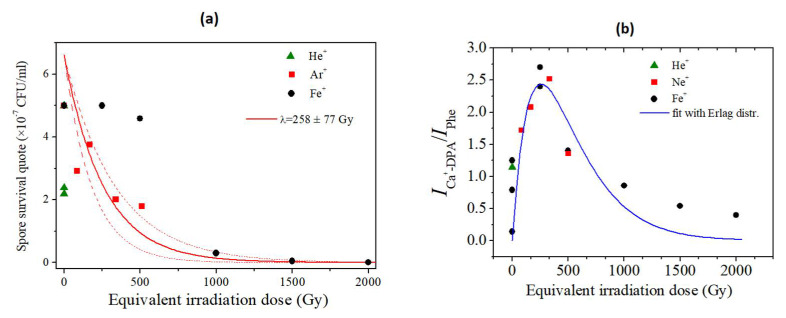
(**a**) Survival fraction of spores irradiated in a water solution (data from Figure 2): comparison of HZE efficacy. Equivalent doses refer to Fe. The exponential decay (red curve) is obtained from the Erlag distribution for k = 0 and λ = 258 ± 77 Gy (see Appendix B). The survival portion of spores is related to the zero-event probability of impact with ions. (**b**) SERS evaluation of the ratio I _Ca2+-DPA_/I _Phe_ as a function of the equivalent irradiation dose Φ refers to Fe (data from Figure 5). This ratio is assumed to be proportional to the Ca^2+^-DPA leaked from spores. The experimental data have been fitted by the Erlag distribution curve, with fit parameters k = 1 and λ = 258 ± 77 Gy (see Appendix B). The germination is activated by a single event of ion impact.

SERS spectroscopy allowed for the detection of the molecular species released by the spores in the outer environment upon irradiation. Such an analysis was performed by measuring the ratio of the intensities of the signal of Ca^2+^-DPA detectable out of the spore vs. the signal due to proteins (as Phe’s signal) on the spore’s surface or potentially released in the medium. The SERS results (Figure 8) are compatible with a double regime response of the spores to the ion radiation process: below a radiation threshold value, the release of Ca^2+^-DPA from the inner core is the main event taking place. Such a phenomenon can be due to a change of inner membrane’s permeability that is also typical of the first stages of the germination process, and that results in the release of Ca^2+^-DPA and ions from the inner core. It could be hypothesized that irradiation below this radiation dose’s threshold value acts as a germination-like process’s stimulator, as previously reported for other stressing factors such as temperature or high pressure [30]. This result is also in accordance with previous studies carried out on estremophilic species that showed that, in some cases, HZE irradiation can act by somehow triggering the germination process of bacterial spores [3]. The dose’s threshold value depended upon the ion used; therefore, an equivalent ion radiation dose (Φ) that takes in account the radiation cross section (see Section 2.6) was introduced. The threshold dose value for the case of Fe ion irradiation is around 258 Gy (Figure 8a). At this dose, the estimated Ca^2+^-DPA level outside reaches its maximum. The maximum release takes place at radiation doses that do not significantly reduce the spores’ viability: in the case of higher LET ions, i.e., Fe and Ar, it was found at doses at which the survival fractions were higher than 90% and 50%, respectively. At doses higher than the threshold, the damages due to the radiation process are larger, as confirmed by the lower survival fraction: in these conditions, the signals detected in the spores’ outside showed a lower Ca^2+^-DPA vs. protein ratio, thus suggesting that the inactivation found at higher doses is accompanied by the release of proteins too from the inner to the outer of spores. This scenario is supported by the fit of the data with the Erlang probability distribution (Appendix B). The assumed probability distribution was built up assuming that, until the threshold level of damage, the number of spores exuding Ca^2+^-DPA increases but, at doses higher than the threshold, the Ca^2+^-DPA exudation process is accompanied by the protein release that is then associated with a nearly total inactivation of the spores.

IR microspectroscopy afforded information about the changes in the spore inner environment upon irradiation and storage. In this case, we measured the ratio of the intensity of Ca^2+^-DPA vs. amide I and II signals of the whole protein pattern, which gave us information about the ratio of dipicolinate retained in the spores’ core vs. the degree of denaturation in the proteins’ secondary structures. At lower radiation doses, the denaturation process of the whole spores’ proteins appears to be the prevailing phenomenon, since the ratio for Ca^2+^-DPA vs. amide I and II protein signals increases with increasing doses; then, there is a pitfall in this ratio, attributable to a higher loss of dipicolinate from the inner core with respect to the loss of proteins integrity. At highest doses, the denaturation of whole spores’ proteins still continues but is finally above the loss of Ca^2+^-DPA. Overall, the inactivation of bacterial spores after ionizing radiation is characterized by the expected loss of integrity of proteins and other fundamental cellular components that takes place in conjunction with the release of Ca^2+^-DPA from the inner core: a similar phenomenon was observed also when irradiation was carried out with gamma rays [7] for which it was not possible to find evidence of a germination-like process, at least in the early stages of inactivation.

## 5. Conclusions

According to some most debated theories about the origin of life on Earth, i.e., *panspermia* and *lithopanspermia*, the first forms of life on our planet could have originated from bacterial species travelling through space that, once arrived on Earth, functioned as an “*inocolum*”. In this frame, cells that resisted the extreme conditions of outer space (absence of water, ionizing radiations, vacuum, etc.), would have been able to settle on Earth and, upon somehow finding favorable environmental conditions, thrive and start growing. One of the most challenging factors of the space environment is represented by exposure to galactic cosmic rays, of which the HZE ions constitute the major cause of lethal effects on cells. In this frame, we investigated the cellular response of two extremophilic species exposed to ionizing radiation by analyzing survival and biochemical changes either soon after the irradiation or after long-term storage.

The results presented here suggest that the exposure conditions, besides the radiation dose and the HZE type, strongly affect the cells’ survival and molecular response. Indeed, cells or spores travelling or staying for a long time in desiccated environments have more chances to survive, while their probability to survive in the presence of water decreases with increasing doses of radiation. Notably, the radiation doses delivered in this study can be absorbed only after a long time in the environment in spsace. As previously reported [24,31], the average value of radiation in the open space is about 0.2–0.5 Gy per year: in our case, as an example, hydrated cells of the halophilic species *H. hispanica*, could probably survive after 3000–7500 years of exposure to Ar HZE, while their resistance could reach 4000–10,000 years if radiation exposure occurs in the anhydrous state. Similarly, bacterial spores in the dry state could survive Fe HZE particle exposure for about 3000–7500 years, while their resistance in a hydrated environment would be limited to 1000–2500 years.

In conclusion, the effect of exposure on ionizing radiations also depends upon the hydration state of the samples, and for such kinds of studies, extremophilic microorganisms are a valuable biological model for astrobiology; nonetheless, future studies need to include exposure to real space conditions and further analysis to gain more insights in the molecular determinants of resistance and adaptation to space conditions.

## Figures and Tables

**Table 1:** HEV ion radiation process parameters.

ion	Energy (MeV/*n*)	LET (keV/µm)	Intensity (pps)	Dose Rate (Gy/min)	Delivered Doses (Gy)/Irradiation’s Duration (min)
He	150	2.2	1.2 × 10^10^	4.2	250/59.5, 1000/82.6
Ar	500	90	2.4 × 10^8^	6.8	250/36.8, 500/73.5, 1000/147.1, 1500/220
Fe	500	200	2.5 × 10^8^	12.1	250, 500, 1000, 1500, 2000

**Table 2:** Normalized ion cross sections.

ion	M_1_(amu)	Z_1_	Normalized σ
He	4	2	4 × 10^−4^
Ar	40	18	0.34
Fe	55.9	26	1

## Appendix B

The Erlang distribution *f* is a particular case of Gamma functions that suitably describe events that can be classified by integer numbers. The Gamma function is widely used for describing stochastic events. The wait-time *ϕ* for the k event of a unidimensional Poisson process with probability 1/λ is given by the following [32]:

(A2) $$f\left(\phi ,k,\lambda \right)=\frac{1}{k!\lambda^{k+1}}\phi^{k}e^{\frac{-\phi }{\lambda }}$$

In our case, *ϕ* represents the ion irradiation dose and the probability distribution *f* is related to the event probability (i.e., the impact of k ions with a spore) for a given irradiation dose.

The fit of the experimental data with the Erlang distribution *f* for the Fe^+^ irradiation process was obtained for λ = 258 ± 77 Gy and a pre-factor value of 1709 ± 476. The parameter k = 0 was fixed for the data reported in Figure 8a (the survival portion of spores is related to the zero-event probability of impact with ions), and k = 1 was fixed for the data in Figure 8b (the onset of a germination-like process is activated by a single event).
